# Prevalence of Periodontal Pathogens in Slovak Patients with Periodontitis and Their Possible Aspect of Transmission from Companion Animals to Humans

**DOI:** 10.3390/biology11101529

**Published:** 2022-10-19

**Authors:** Miriam Sondorová, Ján Kučera, Jana Kačírová, Zuzana Krchová Nagyová, Natália Šurín Hudáková, Tomáš Lipták, Marián Maďar

**Affiliations:** 1 Department of Microbiology and Immunology, University of Veterinary Medicine and Pharmacy in Kosice, Komenskeho 73, 041 81 Kosice, Slovakia; 2 Department of Dentistry and Maxillofacial Surgery, Faculty of Medicine, University of Pavol Jozef Safarik in Kosice, Tr. SNP 1, 040 01 Kosice, Slovakia; 3 Department of Stomatology and Maxillofacial Surgery, Faculty of Medicine, Pavol Jozef Safarik University in Kosice, Tr. SNP 1, 040 11 Kosice, Slovakia; 4 Small Animal Clinic, University Veterinary Hospital, University of Veterinary Medicine and Pharmacy in Kosice, Komenskeho 73, 041 81 Kosice, Slovakia

**Keywords:** periodontitis, periodontal pathogens, humans, *Porphyromonas gulae*, transmission, companion animals

## Abstract

**Simple Summary:**

Periodontal disease represents a worldwide health problem. Human periodontal pathogens such as *Treponema denticola*, *Porphyromonas gingivalis*, *Tannerella forsythia* and *Aggregatibacter actinomycetemcomitans* are the cause of inflammatory response resulting in periodontitis. *Porphyromonas gulae* is mostly involved in periodontitis in dogs; however, it is not a common pathogen in humans. This study deals with the prevalence of periodontal pathogens in Slovak patients with periodontitis. Furthermore, based on the previous findings of animal-to-human transmission of periodontal pathogens, this study also assesses the possible bacterial transmission between animals and their owners. The highest prevalence in Slovak patients amongst the monitored periodontal pathogens had *T. forsythia.* In regard to the limited information available on *T. forsythia*, antibiotic sensitivity of this bacterium was evaluated. Most of the *T. forsythia* isolates were susceptible to antibiotics, namely amoxicillin-clavulanic acid, clindamycin and moxifloxacin, while they were resistant to metronidazole. Moreover, the transmission of *P. gulae* between animals and their owners was confirmed. Based on the similarity of *P. gulae* with human *P. gingivalis*, there arises the question as to whether *P. gulae* can also be involved in the periodontitis pathogenesis in humans. However, more studies are required for further clarification.

**Abstract:**

Oral health and diseases are greatly influenced by oral bacteria. During dysbiosis, bacterial composition changes, which can lead to periodontitis. Periodontitis in humans is associated with periodontal pathogens such as *Treponema denticola*, *Porphyromonas gingivalis*, *Tannerella forsythia* and *Aggregatibacter actinomycetemcomitans*. Animal-to-human transmission of some of these pathogens has also been reported. The aim of this study was to evaluate the prevalence of periodontal pathogens in Slovak patients and to assess the possible risk of transmission of these pathogens from animals to their owners. The presence of periodontal pathogens in dental plaque was monitored by PCR. Amplified products were analysed using Sanger sequencing. *T. forsythia* isolates were assessed for the susceptibility to different antibiotics using the disk diffusion method. In humans, *T. denticola*, *P. gingivalis*, *T. forsythia* and *A. actinomycetemcomitans* were present in 69.23%, 69.23%, 100% and 84.62%, respectively. Most isolates of *T. forsythia* were susceptible to amoxicillin-clavulanic acid, clindamycin and moxifloxacin, but they were resistant to metronidazole. The transmission of *T. forsythia* from animals to their owners was not proven based on sequence analysing. On the other hand, transmission of *Porphyromonas gulae* was confirmed, but the risk of its involvement in the pathogenesis of periodontitis in humans must be further investigated.

## 1. Introduction

The oral cavity provides a habitat for a diverse bacterial community comprising hundreds of bacterial species that exist in homeostatic balance with their host [1]. Oral bacteria colonize hard and soft tissues in the oral cavity and play a significant role in influencing oral health and disease [2]. Compared to other tissues, the teeth provide a hard surface where the greatest accumulation of bacteria occurs due to better attachment, with the formation of dental plaque, also known as dental biofilm [3]. In general, various endogenous and exogenous factors are involved in the dynamics of the composition of the oral microbiota. In a state of dysbiosis, there are changes in this bacterial composition and disruption of the homeostatic balance, which can ultimately lead to oral and even systemic diseases [4]. One of the most common diseases associated with oral bacterial dysbiosis is periodontitis, which represents a worldwide health problem [5].

Periodontitis is characterized as a chronic multifactorial inflammatory disease affecting the supporting tissues surrounding the teeth. Various inflammatory manifestations can initially lead to progressive tissue destruction, followed by alveolar bone involvement and finally tooth loss [6,7]. This disease is associated with intermittent periods of remission and relapse [5]. On the basis of clinical manifestation, periodontitis was previously classified into chronic and aggressive form [8]. Due to limited differences, e.g., in the microbial composition or lack of specific immune inflammatory response patterns, these forms of periodontitis were grouped into one category under the name periodontitis [9]. It was divided into four stages based on severity and complexity of disease management and three grades according to the progression risk of disease [10].

Complex interactions between the multispecies bacterial community in dental plaque and the host’s immune response play a role in the etiopathogenesis of periodontitis [11]. The presence of specific bacterial pathogens, such as *Treponema denticola*, *Porphyromonas gingivalis* and *Tannerella forsythia* belonging to the red complex, or *Aggregatibacter actinomycetemcomitans*, is strongly associated with an increased risk of periodontitis [12]. During periodontitis, there is also a reduction or absence of some commensal bacterial species in favour of these periodontal pathogens [13]; therefore, a qualitative and quantitative bacterial difference can be observed between patients with and without periodontitis [14]. Moreover, from an epidemiological point of view, the prevalence and distribution of periodontal pathogens may be different in relation to various geographical locations and populations [15].

Periodontitis is also frequently seen in veterinary practice [16]. While it is affecting approximately 30% of adult human population, there is 80% prevalence in dogs older than 2 years [17] and 70% prevalence in cats of the same age [18]. Amongst pathogenic bacteria of companion animals there are *T. denticola*, *Porphyromonas gulae*, *P. gingivalis*, *T. forsythia*, *Prevotella intermedia*, *Prevotella nigrescens*, and *A. actinomycetemcomitans* [19]. It has been reported that some of these periodontal pathogens can be transmitted from companion animals to their owners, especially through close physical contact [20,21]. Approximately 16.4% of bacterial species of canine subgingival plaque were shared with the human population [22]. Such examples include *P. gulae*, *T. forsythia*, and *Campylobacter rectus* [23].

Based on the above, the present study was carried out to provide insights on the prevalence of periodontal pathogens in Slovak patients with periodontitis. Moreover, the risk of possible transmission of periodontal pathogens from companion animals to owners was evaluated in some patients, as well.

## 2. Materials and Methods

### 2.1. Collection of Plaque Samples from Human and Animal Population

Thirteen Slovak patients with periodontitis were included in this study. The patients were non-smokers, had more than 20 teeth and had not undergone mechanical periodontal therapy before sampling. None of them had used any nonsteroidal anti-inflammatory drugs, antibiotics or immunosuppressive drugs within the last 6 months. According to the new classification system [24], the participants of this study were assigned the appropriate stage and grade of periodontitis. Clinical examinations, assessment of periodontal status and collection of dental plaque samples were carried out by two professional practitioners. A plaque sample was taken individually from each patient from the places where the deepest periodontal pockets were present, mainly between the first and the second molars, using a sterile curette. In addition, screening of patients’ genotype was performed within three genetic variants (rs1800587, rs1143634 and rs419598) in the gene cluster for interleukin-1 (IL-1) and gene encoding the human leukocyte antigen (HLA)-DR4. Samples were taken by inserting special sampling sticks into the gingival sulcus for 10 s. These samples were sent for DentalGen genetic testing using real-time PCR (GHC GENETICS SK, Bratislava, Slovakia).

From companion animals, samples were taken from a total of five animals (1 cat and 4 dogs) that lived in the same household with some patients, using a sterile syringe needle. The sample included dental plaque from all types of teeth, especially from the upper dentes canini and dentes premolares. Before sampling, an intraoral examination was performed in non-anesthetized animals, during which the stage of periodontitis was evaluated according to Bauer et al. [25]. These examinations were carried out by a veterinarian at the Small Animal Clinic, University of Veterinary Medicine and Pharmacy in Kosice.

Dental plaque samples from humans and animals were collected into the microtubes containing 300 µL of phosphate-buffered saline (PBS). Then, samples were vortexed at maximum speed for 20 s and shaken at 1400 rpm (Thermoshaker TS-100C, BioSan, Riga, Latvia) for 5 min to homogenize the contents. A volume of 50 µL was taken from the homogenized sample into bacterial glycerol stocks (growth medium and glycerol in equal volume 1:1). Plaque samples in PBS and bacterial glycerol stocks were stored at −70 °C until processing.

### 2.2. Ethical Approval

The Ethics Committee of the Faculty of Medicine at the Pavol Jozef Safarik University in Kosice and Ethics Committee of Louis Pasteur University Hospital in Kosice approved the study (protocol code 2021/EK/11068). The participants were fully informed about the purposes and procedures of study and provided informed consent in accordance with the Declaration of Helsinki.

The study protocol involving companion animals was approved by the Ethics Committee of the University of Veterinary Medicine and Pharmacy in Kosice (protocol code EKVP/2022-01). The owners of the animals provided informed consent.

### 2.3. Extraction of Microbial DNA

Plaque samples were thawed before DNA extraction, centrifuged at 10,000× *g* for 10 min at 4 °C, and then the supernatant was removed. A modified protocol for phenol-chloroform DNA extraction was used [26]. In brief, 180 µL of lysis buffer (50 mM Tris-HCl, 10 mM EDTA and 1% sodium dodecyl sulfate) and 25 µL of proteinase K (Roche Diagnostics GmbH, Mannheim, Germany) were added to the pellets. The microtubes were incubated at 55 °C for 2 h, with shaking at 300 rpm (Thermoshaker TS-100C, BioSan, Riga, Latvia). Proteinase K was inactivated by heating at 95 °C for 5 min. Subsequently, they were centrifuged at 10,000× *g* for 5 min at 23 °C and the supernatant was transferred to the new microtubes. Phenol and chloroform were added to the supernatant in an equal volume of 1:1 and centrifuged at 10,000× *g* for 5 min at 23 °C. The upper aqueous phase was transferred to the new microtube. Isopropyl alcohol (0.6 volume of supernatant) and 3 M sodium acetate solution (0.1 volume of supernatant) were then added. The nucleic acids in the mixture were allowed to precipitate overnight at 4 °C. The following day, DNA was pelleted at 10,000 × g for 10 min at 4 °C, washed with 100 µL of cold 75% ethyl alcohol and dried at 35 °C for 5–10 min. The pellets were resuspended in 30 μL of TE buffer (50 mM Tris-HCl and 10 mM EDTA). The quality and quantity of the extracted DNA were analysed by spectrophotometry using NanoDrop 1000 (Thermo Fisher Scientific, Waltham, MA, USA). According to the measured DNA concentration, samples were diluted with molecular grade water to a concentration of 50 ng/µL template DNA, and the thus diluted samples were stored at −70 °C until use.

### 2.4. Molecular Detection of Periodontal Pathogens

PCR amplification was carried out in a thermal cycler (TProfessional Basic, Biometra GmbH, Göttingen, Germany). The reaction mixture (50 µL) contained 2 µL (100 ng) of sample (DNA template), OneTaq 2X Master Mix with standard buffer (New England Biolabs, Foster City, CA, USA), molecular water and primers at concentration of 33 µM. The primers used for PCR amplification of *Treponema* species, *T. denticola*, *P. gingivalis*, *P. gulae*, *T. forsythia* and *A. actinomycetemcomitans*, and thermocycling conditions are listed in Table 1. Negative controls (RNAse-free water) were included for each set of primers in each of the PCR reactions.

Amplification products were subjected to electrophoresis in 2% agarose gel and visualized with GelRed (Biotium, Inc., Hayward, CA, USA) under UV light [31]. A 100 bp DNA ladder was used as a molecular weight marker (New England Biolabs, Foster City, CA, USA).

### 2.5. Sequencing and Data Processing

The amplification products of all PCR reactions except *A. actinomycetemcomitans* were sent for Sanger sequencing from one or both directions (Microsynth, Wien, Austria). Homology searches with sequences were performed by online nucleotide BLAST (Basic Local Alignment Search Tool) algorithm (https://blast.ncbi.nlm.nih.gov/Blast.cgi, accessed on 4 August 2022). In the case of *flaB2* gene, the nr/nt database was used, and for the 16S rRNA gene, both the nr/nt and 16S rRNA databases were used. The chromatograms of sequences were edited and aligned using Geneious alignment in Geneious 8.0.5 (Biomatters, Auckland, New Zealand), and phylogenetic trees were constructed for *P. gingivalis*, *P. gulae* and *T. forsythia* using MUSCLE Alignment and Neighbor-joining in Geneious 8.0.5. The nucleotide sequences were deposited in GenBank under accession numbers OP678047–OP678083.

### 2.6. Disk Diffusion Susceptibility Test

In vitro antibiotic susceptibility tests for *T. forsythia* were performed due to the limited information available on this bacterium. From bacterial glycerol stocks containing dental plaque from humans and animals, suspensions in a volume of 25–50 µL (based on the amount of dental plaque acquired) were inoculated onto Tryptic Soy Agar (TSA) supplemented with 5% sterile horse blood, N-acetyl muramic acid (NAM, 1 mg/L; Sigma-Aldrich, St. Louis, MO, USA) and hemin (5 mg/L; Sigma-Aldrich, St. Louis, MO, USA). Plates were incubated anaerobically (BBL GasPak™ Plus, Becton, Dickinson and Co., Franklin Lakes, NJ, USA) for 7 days at 37 °C [1]. Based on typical morphology and Gram staining, colonies were selected and cultured in an anaerobic environment for 72 h at 37 °C. For final identification of *T. forsythia*, DNA was isolated from pure bacterial cultures through DNAzol Direct (Molecular Research Center Inc., Cincinnati, OH, USA) according to the manufacturer’s instructions and verified by the previously mentioned PCR. Antibiotic sensitivity was determined by disk diffusion on TSA-NAM. Bacterial suspensions were prepared from pure cultures and turbidity of suspensions was adjusted to 0.5 McFarland in saline solution at 565 nm wavelength (DEN-1 McFarland densitometer, Biosan, Riga, Latvia). Subsequently, 100 µL of suspensions were inoculated onto TSA-NAM. Four antibiotic discs (HiMedia, Mumbai, India) namely amoxicillin-clavulanic acid (30 μg), clindamycin (10 μg), moxifloxacin (5 μg) and metronidazole (5 μg) were placed onto the surface of the agar plates within 15 min of inoculation. Then, the plates were incubated anaerobically for 72 h at 37 °C. The inhibition zones were measured and interpreted according to Dubreuil et al. [32] and Nagy et al. [33].

## 3. Results

### 3.1. Baseline Characteristics of the Participants and Companion Animals

Participant’s characteristics are summarized in Table 2. A total of 13 patients in the same gender ratio were ranged in age from 34–68 years, with a mean age of 50.5 ± 10.5 years. Stage I periodontitis was present in six people, while stage II was present only in one patient and stage III in three patients. In three cases, there was periodontitis in between stages II and III. Periodontitis with grade A, grade B and grade C was present in five, six, and two patients, respectively. Most of them regularly underwent dental examinations along with dental hygiene, except patients H5 and H9. Furthermore, five of them were owners of some animal, whereas patients H12 and H13 were a married couple with one dog.

Within genotyping to reveal an increased risk of immune reactivity associated with rapid progression of periodontitis, patients H1, H2, H3, H8, H9 and H10 were found to have a positive genotype within the polymorphism in the genes *IL1-A* (rs1800587), *IL1-B* (rs1143634) and *IL-1RN* (rs419598). On the other hand, patients H5 and H11 had an increased risk of periodontitis progression due to the presence of polymorphism in gene encoding HLA-DR4. Lastly, patient H6 had a positive genotype within gene cluster for IL-1 and in gene encoding HLA-DR4. Patients H4, H7, H12 and H13 had a negative genotype in regards to the polymorphism in genes mentioned above.

Companion animals of different breed and age were assigned periodontal status based on evaluation of clinical signs such as gingivitis, amount of dental plaque, presence of dental calculus or tooth loss (Table 3). Most of the dogs had mild gingivitis, except for dog 4 that showed presence of only dental plaque without gingivitis. None of the animals have been castrated or sterilized and their teeth had not been previously treated before sampling.

### 3.2. Detection of Periodontal Pathogens in Humans

Using species-specific PCR for detection of *T. denticola*, this pathogen was present in 69.23% of dental plaque. Similarly, *P. gingivalis* was detected in 69.23%, while *P. gulae* was found only in 23.08%. All the plaque samples in patients were positive for the presence of *T. forsythia*. *A. actinomycetemcomitans* was confirmed in most patients (84.62%) except H5 and H7. Prevalence of periodontal pathogens in plaque samples of patients is listed in Table 4. After sequencing the amplified products of PCR using primers for *Treponema* sp., these primers were found to be inappropriate for the detection of *T. denticola*, since all the samples with amplified products were identified as *Treponema vincentii* with 100% homology. On the other hand, based on BLAST analysis all PCR products using primers for the detection of *T. denticola* were identified as *T. denticola* showing 100% homology. The primers targeting *P. gingivalis* were not found to be species-specific, since after sequencing the PCR products, there was also confirmation of other bacterial species, namely *P. gulae* and *Capnocytophaga* sp. with 100% and 97.48% homology, respectively, in patients H4 and H8. Besides that, all the other amplified products showed homology ranging from 99.67% to 100% with *P. gingivalis*. Using species-specific primers for *P. gulae*, all positive PCR products after sequencing showed 100% homology with *P. gulae*. Lastly, for *T. forsythia* all sequences homology ranged from 99.3% to 100%.

### 3.3. Comparison of Periodontal Pathogens between Companion Animals and Their Owners

In the case of companion animals, only species-specific primers were used for the identification of *T. denticola*. Its presence was confirmed in one dog (H10D3). *P. gulae* was detected in all animals using both primer pairs for *P. gulae* and *P. gingivalis*, which was subsequently confirmed by sequencing (homology ranging from 99.58% to 100%). *T. forsythia* and *A. actinomycetemcomitans* were identified in all animals by PCR. The presence of periodontal pathogens in plaque samples of companion animals is listed in Table 5.

**Figure 1:**
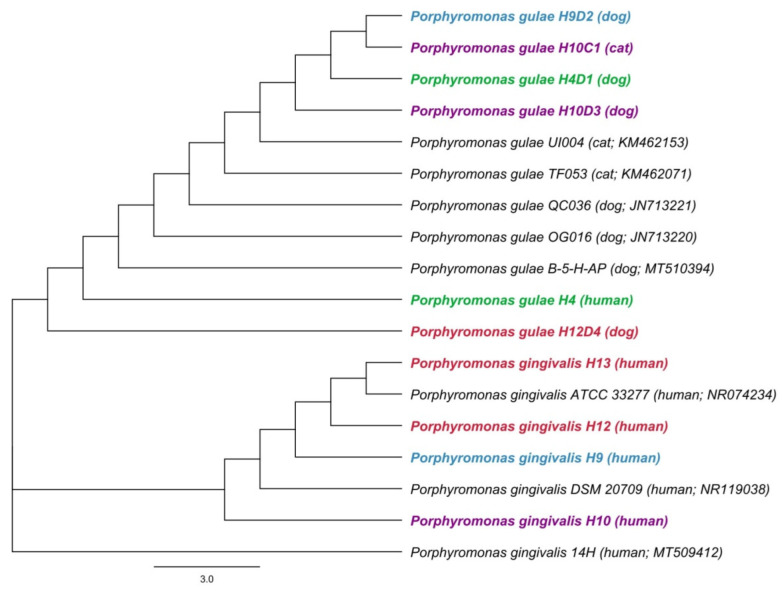
The phylogenetic tree of *Porphyromonas gingivalis* and *Porphyromonas gulae* based on 16S rRNA gene sequences. A phylogenetic tree for sequences obtained using primers for *P. gingivalis* was constructed by the neighbor-joining method. Length of 16S rRNA gene sequence used to construct the phylogenetic tree was 240 bp. Hosts and accession numbers for 16S rRNA gene sequences obtained from the GenBank database are given in parentheses. Each color represents the owners and their animals.

Sequence analysis of the 16S rRNA fragment using both primer pairs for *P. gulae* and *P. gingivalis* showed identical sequences of *P. gulae* in patient H4 and his dog (H4D1). On the other hand, in patient H9 and his dog (H9D2), identical sequences of *P. gulae* were confirmed only using species-specific primers for *P. gulae*. Based on the detection of *P. gulae* in owners and their dogs, there is the possibility of transmission resulting from identical sequences of human and animal strains. Sequences were compared to known *P. gulae* and *P. gingivalis* gene sequences from the sequence database of the National Center of Biotechnology Information (NCBI, Bethesda, MD, USA). A phylogenetic tree for sequences obtained using primers for *P. gingivalis* is shown in Figure 1.

**Figure 2:**
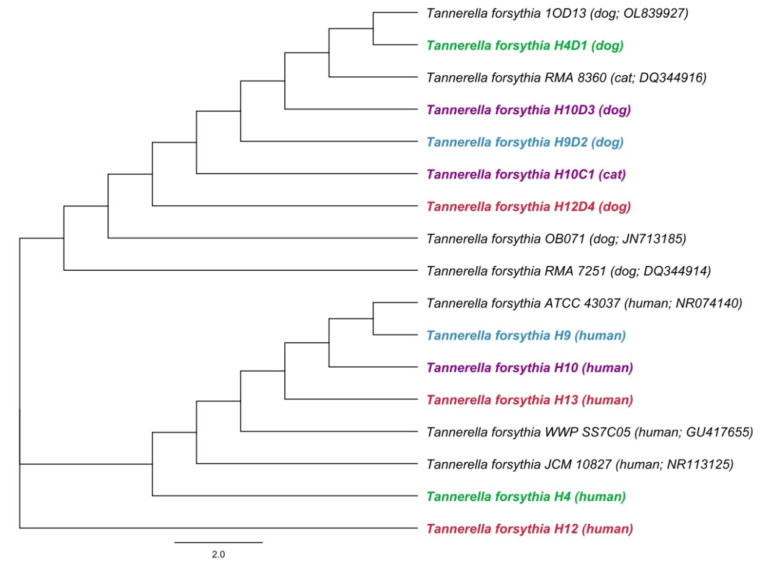
A phylogenetic tree based on comparison of 16S rRNA gene sequences of *Tannerella forsythia*. A phylogenetic tree was constructed by the neighbor-joining method. Length of 16S rRNA gene sequence used to construct the phylogenetic tree was 480 bp. Hosts and accession numbers for 16S rRNA gene sequences obtained from the GenBank database are given in parentheses. Sequences of owners and their animals are distinguished by different colors.

A phylogenetic analysis showed differences between the sequences of the 16S rRNA gene for *T. forsythia* in humans and animals (Figure 2). Based on this, the transmission of *T. forsythia* from companion animals to their owners has not been proven.

### 3.4. Antibiotic Susceptibility of Tannerella forsythia

Regarding antibiotic sensitivity, all *T. forsythia* isolates were susceptible to amoxicillin-clavulanic acid and moxifloxacin (Table 6). Furthermore, all isolates were also found to be susceptible to clindamycin with the exception of H12D4a isolate. Lastly, none of the *T. forsythia* isolates were sensitive to metronidazole. Susceptibility for amoxicillin-clavulanic acid and moxifloxacin was defined if the diameter zone was ≥21 mm, for clindamycin it was ≥25 mm and for metronidazole the diameter zone was ≥15 mm. Despite the fact that *T. forsythia* was identified by PCR in all samples, *T. forsythia* was not isolated from patients H1, H2, H10 and companion animals H4D1, H9D2, H10D3 and H10C1.

## 4. Discussion

Oral diseases continue to be a major global health problem, with periodontitis being the most significant worldwide. Oral health is affected by the presence of predominantly anaerobic Gram-negative bacteria [34]. Interactions of these bacteria with the host can lead to disease; however, most of them can be present even in healthy individuals and coexist in harmony with the host due to the different virulent potential and pathogenicity of the bacterium [35]. Periodontal pathogens such as *P. gingivalis*, *T. forsythia*, *T. denticola*, *P. intermedia* and *A. actinomycetemcomitans* have been widely studied in terms of their virulence factors involved in periodontal pockets colonization or periodontal tissue destruction. It has been shown that the detection rate of *P. gingivalis*, *T. forsythia* and *P. intermedia* is significantly higher in periodontitis patients than in healthy individuals, but it does not significantly differ in between various stages of periodontal disease [36]. Similarly, determining the global distribution of bacterial species related to periodontitis may be important in terms of potential specifics associated with different ethnic and geographic factors [37]. Moreover, different detection methods can affect the capture rate of periodontal pathogens [38]. Among the available methods, PCR methods are considered to have higher sensitivity and specificity in the early detection of these periodontal bacteria [39]. For example, traditional PCR can qualitatively assess the presence of periodontal pathogens, while real-time PCR is commonly used to quantify these pathogens [40]. Using molecular methods, this study analysed the prevalence of five periodontal pathogens of humans and companion animals, namely *T. denticola*, *P. gingivalis*, *P. gulae*, *T. forsythia* and *A. actinomycetemcomitans* in the dental plaque of Slovak patients suffering from periodontitis and their animals.

*T. denticola*, being one of the most resistant bacteria frequently identified in primary dentition infection, is highly associated with periodontal diseases. It is commonly isolated from periodontal pockets of adults and it has great influence on the inflammation by stimulating the production of cytokines and it also causes disruption of tissue homeostasis [41]. Based on that, *T. denticola* is predominantly linked with severe periodontitis. However, based on molecular studies, other oral treponemes, including *T. vincentii*, are also associated with periodontal disease [42]. In our study, *T. denticola* was detected in 69.23% of Slovak patients. Similar findings were shown in the study by Jepsen et al. [43], in which this pathogen was present in 76.5% of German periodontitis patients. In addition, in the study by Feng et al. [44] *T. denticola* was present in 87.8% of Chinese patients with periodontitis and in the study by Torrungruang et al. [45] *T. denticola* was demonstrated in 82% of Thai patients.

Amongst the most important periodontal pathogens *P. gingivalis* appears to be one of the main etiological factors in the pathogenesis and development of inflammatory processes of periodontitis [46]. *P. gingivalis* expresses a wide array of virulence factors which include fimbriae, gingipains, collagenase, lipopolysaccharide, haemagglutinins, capsule, and superoxide dismutase [47]. These factors may cause destruction of the periodontal tissue on their own [48] or they can induce inflammatory reactions in the host and cause impairment of innate immune responses [49]. Our findings show that *P. gingivalis* was present in 69.23% of Slovak patients. Similar results were also presented in German [43] and Indian [50] patients, in which the prevalence was 68.2% and 66%, respectively. In the study by Rafiei et al. [51] the prevalence of *P. gingivalis* in periodontal disease group was 78% and in the study by Mínguez et al. [52] the prevalence in Moroccan periodontitis patients was 84.4%.

*T. forsythia* is also considered to be a part of periodontal oral microbiome [53] and it remains an understudied periodontal microorganism [54]. In a study by Alazemi et al. [55] *T. forsythia* was detected by PCR in 83.3% of Kuwait patients with severe and moderate periodontitis. In the case of the Uruguayan population with periodontitis, *T. forsythia* was among the most widespread species (92%) in the examined periodontal pathogens [56]. Prevalence of *T. forsythia* among Italian patients with periodontitis was 72.7%, with no statistical differences between geographical areas [57]. In this study, *T. forsythia* was detected in all Slovak patients regardless of the stages and grades of periodontitis, which probably indicates its strong association with this disease. Based on practically still limited information about the sensitivity of *T. forsythia* to various antimicrobial substances [54], our study also evaluated the antibacterial sensitivity of selected antibiotics. Amoxicillin-clavulanic acid, clindamycin, moxifloxacin, and metronidazole are among the most well-known drugs used as part of periodontal therapy in practice [46]. In this study, all *T. forsythia* isolates were sensitive to amoxicillin-clavulanic acid and moxifloxacin, while they showed resistance to metronidazole. Similarly, in the study by Ardila et al. [54], isolates of *T. forsythia* were completely susceptible to moxifloxacin, but the isolates showed resistance to amoxicillin and metronidazole in 25.6%. The antibacterial effect of antibiotics on *T. forsythia* showed resistance to metronidazole, amoxicillin and clindamycin in the study by Abdulrazzaq et al. [34]. However, in our study, 90.9% of the isolates were sensitive to clindamycin, which is contrary to the previous study. Similar to our study, *T. forsythia* was sensitive to clindamycin (80%) and amoxicillin-clavulanic acid (100%); however, it was found to be sensitive to metronidazole, which is in contradiction with our study [58].

*A. actinomycetemcomitans* is considered an oral pathogen that colonizes the oral cavity of the human population and is highly associated with rapidly progressive periodontitis [59]. Its role in the initiation and development of periodontitis as a community activist is considered essential for other periodontal pathogens, as it suppresses the initial host response, allowing their proliferation [60]. *A. actinomycetemcomitans* was relatively rare (16.1%) among Italian adults with periodontitis in the study by Tettamanti et al. [57]. In the study by Jensen et al. [61], the prevalence of *A. actinomycetemcomitans* was 13.3% in the adolescent population in Denmark. The detection frequency of *A. actinomycetemcomitans* in subgingival plaque samples collected from periodontitis patients living in Sweden was 26.3% [62]. In contrast to previous studies, our results show a higher rate of prevalence of this bacterium (84.62%) in Slovak patients. Similarly, in the study by Mínguez et al. [52], the detection frequency of *A. actinomycetemcomitans* was 79.5% in periodontitis patients in Morocco.

Host genetic predispositions within a dysregulated immune response may influence the development of periodontitis through susceptibility to oral dysbiosis and improper host response to bacterial infection [63]. Interleukin-1α plays a critical role in protecting the body from bacteria; however, in result it causes bone resorption and tissue damage, as seen in periodontal disease. Based on that, activity of IL-1 is higher in crevicular fluid obtained from inflamed gingival sites [64]. Consequently, genes encoding its production are receiving attention as potential predictors of periodontitis progression [65]. Moreover, HLA also have an importance in immune responsiveness and involvement in antigen recognition of periodontal bacteria [66]. As a result, there is a positive association of periodontitis with the presence of HLA-DR4 allele that is involved in the pathogenesis of periodontitis [67]. Individuals with positive genotype have an increased risk of periodontitis progression compared to the individuals with a negative genotype [68]. In this study, positive genotype within polymorphism in *IL1-A*, and *IL1-B* genes and *IL-1RN* gene encoding the antagonist receptor of IL-1 was recorded in six patients. Polymorphism in genes encoding HLA-DR4 was present in two patients. Presence of genetic variants in gene encoding HLA-DR4 and gene cluster for IL-1 was recorded in one patient. On the other hand, negative genotype of all the genes mentioned was present in four other patients.

In dogs, periodontal disease is mainly associated with the presence of *P. gulae*, which was originally thought to be the animal biotype of *P. gingivalis*; however, it was also found to colonize human oral cavity [69]. *P. gulae* has similar characteristics as *P. gingivalis* [70], which may result in the potential transmission between animals and their owners [23]. It possesses a broad spectrum of pathogenic properties such as lipopolysaccharide, haemagglutinins, fimbriae and proteases [71]. Similar proteolytic activity to *P. gingivalis* was also detected in *P. gulae* that exhibited similar virulence factors as its human counterpart [72]. Besides that, Nomura et al. [73] found that certain FimA types of fimbrial protein of canine *P. gulae* were phylogenetically close to those of *P. gingivalis*. Moreover, Fujiwara-Takahashi et al. [70] found that FimA types I, III, and IV in *P. gingivalis* were close to types A, B, and C, respectively, in *P. gulae* in dogs. Iwashita et al. [74] identified the *fimA* genotypes in feline *P. gulae* isolates, as well. In this study, the presence of *P. gingivalis* was confirmed in four animal owners, but the pathogen was not detected in any dental plaque samples of companion animals. Kwon et al. [75] detected *P. gingivalis* in 7.44% of canine subgingival plaque samples. On the other hand, in our study *P. gulae* was detected in two humans and their respective dogs, and based on their identical sequences, transmission between owner and its dog can be assumed. Presence of *P. gulae* was confirmed in all animal samples. Similar findings were described in the study by Yamasaki et al. [23], in which *P. gulae* was detected in 71.2% of dogs, while it was present only in 20% of the owners. Iwashita et al. [74] also detected *P. gulae* in approximately 90% of feline plaque samples, regardless the periodontal condition. Moreover, in this study, *T. forsythia* was detected in all dental plaque samples, both human and animal. However, transmission between humans and animals was not confirmed due to different taxa occurring in companion animals and their owners, which contradicts another study. Booij–Vrieling et al. [20] found *T. forsythia* in both cats and the owners and hypothesized that cat-to-owner transmission occurred and that cats may be a reservoir of *T. forsythia*. In comparison, the prevalence of *T. forsythia* in the study by Yamasaki et al. [23] was 77.3% in dogs, but only 30.9% in their owners. On the other hand, Kwon et al. [75] identified *T. forsythia* only in 34.23% of canine subgingival plaque. Further studies are needed to understand the possible transmission of periodontal pathogens.

## 5. Conclusions

Within the limitation of a relatively small sample size, this preliminary study clarified the prevalence of periodontal pathogens in Slovak patients with periodontitis. Based on our findings, the plaque samples analysed by molecular methods showed prevalence of *T. denticola*, *P. gingivalis*, *P. gulae*, *T. forsythia* and *A. actinomycetemcomitans* to be 69.23%, 69.23%, 23.08%, 100% and 84.62%, respectively. In addition, a possible owner to animal transmission of *P. gulae* was confirmed based on sequence analysing. However, these findings evoke additional questions for future studies regarding the risk of its involvement in the pathogenesis of periodontitis in humans. Until then, owners should be reminded that companion animals could be carriers of some periodontal pathogens; therefore, it is necessary to avoid close physical contact with animals and ensure personal hygiene compliance.

## Figures and Tables

**Table 1:** Primers and PCR conditions used to detect periodontal pathogens.

Bacterial Species (Gene)	Primer Sequence (5′ to 3′)	PCR Conditions	Length (bp)	Source
*Treponema* sp. (*flaB2* gene)	ACGGYATTTCYTTTATTCAAGTTGC	94 °C 5 min, 45× [94 °C 30 s, 63 °C 30 s, 72 °C 40 s] 72 °C 5 min	471	[27]
	CGAGTCTGTTYTGGTATGCACC			
*Treponema denticola* (fragment of 16S rRNA gene)	TAATACCGAATGTGCTCATTTACAT	95 °C 2 min, 36× [95 °C 30 s, 60 °C 1 min, 72 °C 1 min] 72 °C 2 min	316	[28]
	TCAAAGAAGCATTCCCTCTTCTTCTTA			
*Porphyromonas gingivalis* (fragment of 16S rRNA gene)	AGGCAGCTTGCCATACTGCG	95 °C 5 min, 36× [95 °C 30 s, 60 °C 1 min, 72 °C 1 min] 72 °C 2 min	404	[28]
	ACTGTTAGCAACTACCGATGT			
*Porphyromonas gulae* (fragment of 16S rRNA gene)	TTGGTTGCATGATCGGG	94 °C 5 min, 35× [94 °C 30 s, 58 °C 1 min, 72 °C 30 s] 72 °C 5 min	300	[29]
	GCTTATTCTTACGGTACATTCAYA			
*Tannerella forsythia* (fragment of 16S rRNA gene)	GCGTATGTAACCTGCCCGCA	95 °C 2 min, 36× [95 °C 30 s, 60 °C 1 min, 72 °C 1 min] 72 °C 2 min	641	[28]
	TGCTTCAGTGTCAGTTATACCT			
*Aggregatibacter actinomycetemcomitans* (fragment of 16S rRNA gene)	GCTAATACCGCGTAGAGTCGG	95 °C 3 min, 35× [94 °C 45 s, 55 °C 1 min, 72 °C 1 min] 72 °C 5 min	500	[30]
	ATTTCACACCTCACTTAAAGGT			

**Table 2:** General information about patients and their periodontal status.

Patients	Gender	Age (Years)	Periodontitis		Animals
			Stage	Grade	
H1	F	58	III	B	
H2	M	44	I	B	
H3	M	61	II–III	B	
H4	F	38	I	A	Dog
H5	F	51	I	A	
H6	M	45	III	B	
H7	M	68	II–III	B	
H8	M	63	I	A	
H9	F	63	II	C	Dog
H10	M	41	II–III	B	Dog, Cat
H11	F	34	I	A	
H12	M	46	III	C	Dog
H13	F	44	I	A	Dog

H–human, F–female, M–male.

**Table 3:** Basic information and periodontal status of the companion animals.

Animals	Sample Name	Gender	Age (Year)	Breed	Periodontal Stage
Dog 1	H4D1	M	1.5	Labrador Retriever	I
Dog 2	H9D2	F	10	Vizsla	I
Dog 3	H10D3	F	2	x Siberian Husky	I
Dog 4	H12D4	M	6	French Bulldog	Healthy
Cat 1	H10C1	F	10	European Shorthair	III

F–female, M–male.

**Table 4:** The presence of periodontal pathogens in human dental plaque.

Patients	*Treponema denticola*	*Porphyromonas gingivalis*	*Porphyromonas gulae*	*Tannerella forsythia*	*Aggregatibacter actinomycetemcomitans*
H1	+	+		+	+
H2	+	+		+	+
H3	+	+		+	+
H4		+ *	+	+	+
H5				+	
H6	+		+	+	+
H7	+	+		+	
H8		+ **		+	+
H9		+	+	+	+
H10	+	+		+	+
H11	+	+		+	+
H12	+	+		+	+
H13	+	+		+	+

* identified after sequencing as *Porphyromonas gulae*, ** identified after sequencing as *Capnocytophaga* sp.

**Table 5:** The presence of periodontal pathogens in dental plaque of companion animals.

Animals	*Treponema denticola*	*Porphyromonas gingivalis*	*Porphyromonas gulae*	*Tannerella forsythia*	*Aggregatibacter actinomycetemcomitans*
Dog 1		+ *	+	+	+
Dog 2		+ *	+	+	+
Dog 3	+	+ *	+	+	+
Dog 4		+ *	+	+	+
Cat 1		+ *	+	+	+

* identified after sequencing as *Porphyromonas gulae*.

**Table 6:** The antibiotic sensitivity of *Tannerella forsythia* isolates.

Isolate	Inhibition Zone of Antibiotics (mm)			
	AMC	CD	MO	MT
H3c	35	33	30	0
H4a	45	31	25	0
H5a	42	31	30	0
H6a	42	34	27	0
H7b	36	29	30	0
H8b	33	29	27	0
H9b	41	36	29	0
H11a	30	27	25	0
H12a	28	26	22	0
H13b	31	26	31	0
H12D4a	30	0	22	0

AMC–amoxicillin-clavulanic acid, CD–clindamycin, MO–moxifloxacin, MT–metronidazole.

## Data Availability

Not applicable.
